# Bullous Systemic Lupus Erythematosus Successfully Treated With Intravenous Immunoglobulin and Mycophenolate Mofetil

**DOI:** 10.7759/cureus.45800

**Published:** 2023-09-22

**Authors:** Kevin W Chow, Jaleel Jerry G Sweis, Diala Alawneh, Pim Jetanalin, Christian Ascoli, Stephanie Kuschel, Sheryl Hoyer, Marylee Braniecki, Nadera Sweiss

**Affiliations:** 1 Internal Medicine, University of Illinois at Chicago, Chicago, USA; 2 Internal Medicine, University of Jordan School of Medicine, Amman, USA; 3 Rheumatology, University of Illinois at Chicago, Chicago, USA; 4 Pulmonary and Critical Care Medicine, University of Illinois at Chicago, Chicago, USA; 5 Dermatology, University of Illinois at Chicago, Chicago, USA; 6 Pathology, University of Illinois at Chicago, Chicago, USA

**Keywords:** fever with rash, vesiculobullous rash, skin blister, mycophenolate mofetil, cutaneous lupus erythematous, bullous systemic lupus erythematosus (bsle), intravenous immunoglobulin (ivig), systemic lupus erythematosis

## Abstract

Bullous systemic lupus erythematosus (BSLE) is a rare autoimmune blistering disorder of cutaneous lupus erythematosus (CLE) that typically manifests as an acute vesiculobullous eruption in a patient with systemic lupus erythematosus (SLE). Also, it can rarely present as the initial clinical manifestation of SLE. There is no established US Food and Drug Administration (FDA) therapy for BSLE. We report a case of a 71-year-old Hispanic woman with SLE and lupus nephritis classes III and V who presented to the hospital with a worsening rash with painful, ruptured blisters involving the upper arms, chest, and back. Our patient did not respond to topical or systemic steroids but improved rapidly to combination therapy with intravenous immunoglobulin (IVIg) and mycophenolate mofetil (MMF).

## Introduction

Bullous systemic lupus erythematosus (BSLE) is a rare autoimmune presentation of cutaneous lupus erythematosus (CLE) whose diagnostic criteria were first described by Camisa and Sharma in 1983 [1] and updated in 1988 [2]. These criteria include (1) diagnosis of systemic lupus erythematosus (SLE) by American College of Rheumatology criteria; (2) vesicles and/or bullae arising upon but not limited to sun-exposed skin; (3) histopathology compatible with dermatitis herpetiformis including leukocytoclastic vasculitis of the superficial and mid dermis; (4) indirect immunofluorescence which can be negative or positive for circulating autoantibodies against the basement membrane zone (BMZ) via the salt-split skin technique; (5) direct immunofluorescence with IgG and/or IgM and often IgA at the BMZ [1,2]. There is no established US Food and Drug Administration (FDA) therapy for BSLE, although numerous case reports have documented successful resolution with treatment regimens including dapsone, steroids, and various immunosuppressants. Here we present a case of BSLE with systemic lupus flare which responded to intravenous immunoglobulin (IVIg) and mycophenolate mofetil (MMF).

## Case presentation

A 71-year-old woman with SLE, lupus nephritis classes III and V, Sjogren’s disease, and hypertension presented to the hospital with a worsening diffuse rash that had started two months prior. She had scaly pruritic macules with painful, ruptured blisters involving the upper arms, chest, and back. Associated rheumatological symptoms included Raynaud’s phenomenon and photosensitivity. A review of systems was significant for fever, chills, headache, dyspnea, cough, nausea, vomiting, and diarrhea.

She was diagnosed with SLE 15 years prior for which she took hydroxychloroquine (HCQ). She developed nephrotic-range proteinuria two years prior, which was confirmed by biopsy to be lupus nephritis class III and V. The patient was started on 60mg prednisone and MMF 500mg twice daily. During clinic follow-up, the prednisone dose was gradually tapered, MMF dose was up-titrated with the improvement of proteinuria; however, the patient was subsequently lost to follow-up.

Upon admission for BSLE, she was not on corticosteroids, had self-discontinued MMF due to nausea and diarrhea, and was taking only HCQ 200mg daily.

**Figure 1:**
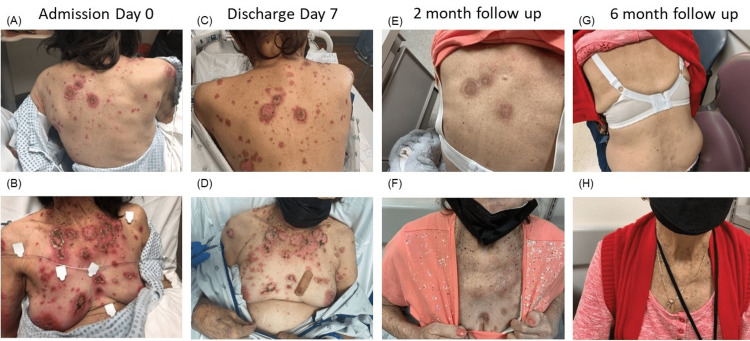
Progression of skin lesions at presentation and after treatment (A, B) Presentation on admission Day 0. (C, D) Lesions after treatment with steroids, IVIg, and MMF at discharge Day 7. (E, F) Residual erythema on oral steroids and MMF maintenance therapy at two-month follow-up. G, H. Complete resolution of lesions at the six-month follow-up.

On examination, the patient appeared ill and was febrile (100.6°F). There was abdominal tenderness and 2+ pitting edema up to the calves bilaterally. She was noted to have diffuse erythematous plaques, with eroded lesions involving the ears, nasal passageway, vermilion lip, back, chest, and R upper leg (Figure 1A) on skin exam. The chest had intact and ruptured bullae with hemorrhagic crusting (Figure 1B). There were coalescing, erythematous macules with telangiectasias on bilateral palms, fingers, plantar feet, and toes. There was no oral or ocular involvement. Labs were significant for proteinuria, elevated CRP, decreased complements, and autoantibodies as summarized in Table 1.

**Table 1:** Laboratory data ^a^ 9 months prior to admission. ^b^ 4 months prior to admission. ^c^ 2 months prior to admission. ^d^ 2 months after discharge.

Variable	Reference Range	Admission	6th-month follow-up
Hematology			
Hematocrit (%)	35.0 - 49.0	34.3	33.3
Hemoglobin (g/dl)	11.7 - 16.0	11.9	11.2
Mean corpuscular volume (µm³)	80.0 - 99.0	95.2	96.6
White-cell count (per mm³)	3,900 - 12,000	3,200	10,000
Platelets	150,000 - 400,000	158,000	277,000
ESR (mm/hour)	<20	88	83
Differential count (%)			
Neutrophils	40.0 - 70.0	67.9	85.4
Lymphocytes	25.0 - 45.0	22.1	12.1
Monocytes	2.0 - 12.0	9.2	2
Eosinophils	0.0 - 6.0	0	0.3
Basophils	0.0 - 2.0	0.9	0.2
Chemistry			
Blood urea nitrogen (mg/dL)	6 - 20	32	19
Creatinine (mg/dL)	0.40 - 1.20	0.99	0.56
Total protein (g/dL)	6.0 - 8.0	5.7	6.3
Albumin (g/dL)	3.4 - 5.0	1.9	3.2
Urine protein creatinine ratio	<0.2	2.32	2.57
Immunologic tests			
IgA pemphigus antibody	<1:10	Negative	
IgG bullous pemphigoid 180 antibody (U/mL)	<9	4	
IgG bullous pemphigoid 230 antibody (U/mL)	<9	2	
Anti-double-stranded DNA antibody (IU)	<24	102	14
Anti-Jo-1 antibody IgG (AU/mL)	0-40	Not detected	
Anti-Ro52 antibody (AU/mL)	0-40	54	
Anti-Ro60 antibody (AU/mL)	0-40	91	
Anti-RNP antibody (AU/mL)	Negative	Negative	
Anti-Smith antibody (AU/mL)	0-19	81	
Scl 70 (AU/mL)	<29	26^a^	
Anti-cardiolipin IgA (APL)	0-11	19^b^	
Anti-cardiolipin IgG (GPL)	0-14	<10^b^	
Anti-cardiolipin IgM (MPL)	0-12	12^b^	
B2Glycoprotein 1 IgG (SGU)	0-20	4^b^	
B2Glycoprotein 1 IgM (SMU)	0-20	93^b^	
B2Glycoprotein 1 IgA (SAU)	0-20	>150^b^	
CMV DNA	Negative	Negative	
Gliadin IgA	<19	20^c^	17^d^
Gliadin IgG	<19	1^c^	2^d^
CRP (mg/L)	<8	14.0	20.2
RPR Titer	<1:1	1:1	
C3 complement (mg/dL)	90 - 180	43	93
C4 complement (mg/dL)	12 - 47	<8	12

**Figure 2:**
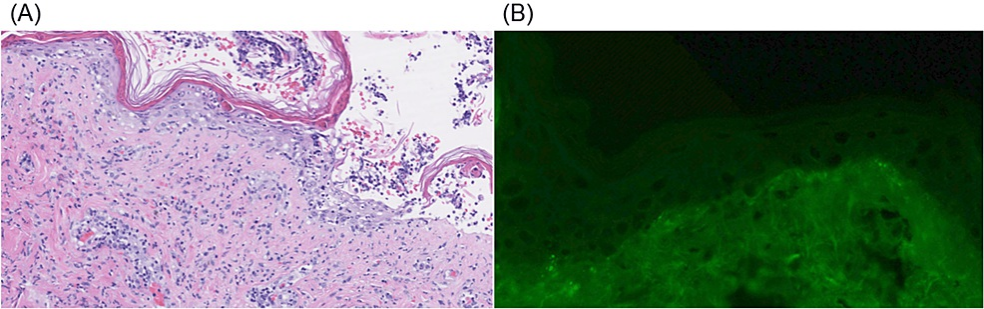
Microscopic appearance of bullous skin lesion (A) Blister with vacuolar interface dermatitis, scattered apoptotic keratinocytes in a thinned epidermis, underlying neutrophils and lymphocytes, extravasated stromal RBCs, and interstitial neutrophils with hematoxylin and eosin stain. (B) 2+ IgG deposition along the follicular basement membrane with direct immunofluorescence

The skin biopsy revealed subepidermal bullae with vacuolar interface dermatitis and scattered apoptotic keratinocytes in the atrophic epidermis. This was accompanied by underlying neutrophils, lymphocytes, and extravasated stromal RBCs (Figure 2A). Direct immunofluorescence of a perilesional lesion demonstrated IgG, IgM, and C3 immuno-deposition along the dermal-epidermal junction and basement membrane of the follicle (Figure 2B). 

The patient’s skin lesions did not respond to topical steroids, oral prednisone 40mg daily, or IV methylprednisolone 50mg twice daily. The overall clinical picture was complicated by acute kidney injury and significant proteinuria suggesting active lupus nephritis, and symptomatic shortness of breath due to bilateral pleural effusions. There was also a concern for possible CMV enteritis due to fevers, persistent GI symptoms, a history of steroid use, and elevated CRP. Therefore, the decision was made to give IVIg 1mg/kg once over one day without dapsone due to GI symptoms and inability to tolerate oral intake. IVIg infusion was tolerated well with no side effects. Improvement of the skin lesions was observed four days after the administration of IVIg (Figures 1C-1D), and dapsone was not started due to rapid improvement. When her GI symptoms subsided, MMF was initiated for treatment for lupus nephritis. The patient was discharged seven days after admission with skin lesions healing on MMF 500mg twice daily, HCQ 200mg twice daily, and 40mg prednisone daily.

At the three-month follow-up, the patient was continued on MMF 500mg twice daily, HCQ 200mg twice daily, and 20mg prednisone daily. The patient tolerated the treatment well. Her skin lesions resolved with residual erythema and scarring (Figures 1E-1F). Between the three- and six-month follow-up visits, the patient had a COVID infection for which she was briefly hospitalized during which her steroid and immunosuppressant regimen was continued. Six months after discharge, medications were 1000mg of MMF three times daily, 200mg HCQ twice daily, and 10mg of prednisone daily, with no nausea or diarrhea and no erythema or rash (Figures 1G-1H).

## Discussion

BSLE is a blistering disorder that appears similar clinically and histologically to other immune-mediated blistering disorders. Dermatitis herpetiformis presents with similar vesiculobullous lesions that are characteristically distributed on extensor surfaces, face, scalp, shoulders, back, and buttocks. Microscopically, there are subepidermal neutrophils, but with granular deposits of IgA and C3 specifically, around the dermal papillae on DIF, and a negative IIF. Epidermolysis bullosa acquisita (EBA) is a non-inflammatory bullous disease with fragile, trauma-induced blisters occurring over non-inflamed or previous scarred skin, although an inflammatory subtype does exist. EBA is known to be a chronic disease, whereas BSLE is more likely to regress completely with treatment [3].

In our case, lesional skin biopsy demonstrated a subepidermal blister accompanied by evident basilar vacuolar changes, lymphocytes, and neutrophils. The papillary dermis showed scattered neutrophils, leukocytoclasia, and stromal extravasated red blood cells. The perilesional skin under direct immunofluorescence revealed immunoreactant deposition of IgG, IgM, IgA, and complement C3 along the dermal-epidermal and follicular basement membranes. Fibrinoid necrosis and vascular immunoreactant deposition were not seen, excluding vasculitis.

Per the literature review, dapsone, alone or in combination with other immunosuppressants (such as azathioprine, cyclosporine, or methotrexate), has been shown to improve BSLE lesions in over 90% of patients with an efficacy of 94% when utilized as monotherapy. However, dapsone is not benign, with treatment being stopped for side effects of hepatitis, anemia, and hypersensitivity syndrome in 23% of cases [4]. Additionally, previous publications suggest corticosteroids and/or immunosuppressive drugs for systemic complications of SLE, and many cases of BSLE respond to these treatments with or without dapsone [3].

Based on the creatinine trend prior to admission, it is likely that lupus disease activity had been increasing, but the patient was lost to follow-up. BSLE is described to be concomitant with lupus nephritis in 50% of cases and cytopenia in 45% [4]. By the time of admission, our patient was in a full lupus flare. With regard to treatment, the patient was first treated with steroids. However, even with escalating doses and intravenous administration for three days, the skin lesions failed to improve, thus IVIg was added for systemic effectiveness and concern for infection. Systemic steroids are known to have an onset of action three to eight hours after administration, therefore this was an adequate trial with treatment failure, and the patient’s clinical improvement can be attributed to the effect of the IVIg [5].

Dapsone was initially withheld due to inability to tolerate oral intake because of nausea and vomiting, and ultimately not given to avoid any potential side effects since the patient had already improved significantly with IVIg. MMF was then resumed, as this had been effective in this patient for lupus nephritis in the past when taken consistently. MMF is well established as first-line therapy for lupus nephritis, and has been successful in treating BSLE in previous case reports, especially when used in conjunction with steroids and other immunosuppressants [4,6]. IVIg 2g/kg/month given over four days for three months has been used in patients with CLE with a decrease in disease activity and relapse rate [7]. Additionally, IVIg 2g/kg/month over five days for one to eight months has been shown to decrease disease activity with good or excellent responses in meta-analyses of other manifestations of SLE including serositis, cytopenias, and lupus nephritis [8]. The mechanism of action of IVIg is thought to be multimodal, including inhibition of innate and adaptive immune cells, suppression of inflammatory cytokines, enhancement of anti-inflammatory mediators, and blockage of antigen-presenting cells. IVIg therapy is relatively safe, with most adverse effects attributable to local injection site reactions. IVIg is frequently used as a second-line therapy for patients with refractory disease or disease relapse, although combination therapy with first-line therapies has shown promise due to its favorable safety profile and synergistic effects [9].

In the few published case reports of BSLE treated with IVIg, four of six cases that received IVIg as adjuvant therapy had successful resolution, with highly variable doses ranging from 2g/kg over three days once, to 0.5 g/kg over four days every four weeks for 14 cycles [4, 10-14]. One case of ineffective IVIg usage did not include the age or specific treatment regimen of the patient [4]. Of the remaining five cases, four cases were pediatric. Dapsone was utilized with IVIg in three cases. One case utilized IVIg with dapsone after the failure of immunosuppressants, a second case utilized IVIg after treatment failure with oral prednisone and dapsone, while the third case used steroids, HCQ, and dapsone but switched to IVIg due to hemolysis [11-13]. Of the remaining two cases, one case avoided dapsone in favor of steroids, HCQ, IVIg, and MMF due to concerns of cytopenia, with symptom resolution. In the last case, dapsone was unable to be obtained, while pulse dose steroids, cyclosporine, HCQ, and IVIg failed, but belimumab was effective [10,14].

## Conclusions

This case highlights IVIg as a treatment option that should be considered for BSLE, especially when accompanied by symptoms of systemic lupus flare or if there are contraindications for dapsone (such as G6PD deficiency) or other immunosuppressive therapy or in the setting of active infection. Case reports have shown IVIg to be effective when used with steroids and other immunosuppressants with or without dapsone, but further controlled studies comparing IVIg to alternative treatments are needed. 
